# 24-Epibrassinolide confers zinc stress tolerance in watermelon seedlings through modulating antioxidative capacities and lignin accumulation

**DOI:** 10.7717/peerj.15330

**Published:** 2023-05-09

**Authors:** Xuefang Liu, Quanwen Zhu, Wentao Liu, Jun Zhang

**Affiliations:** 1 Yangzhou Polytechnic College, Yangzhou, China; 2 Jiangsu Safety&Environment Technology and Equipment for Planting and Breeding Industry Engineering Research Center, Yangzhou, China

**Keywords:** Antioxidant defense, 24-epibrassinolide, Lignin, Oxidative damage, Watermelon, Zinc stress

## Abstract

Zinc (Zn) is an important element in plants, but over-accumulation of Zn is harmful. It is well-known that brassinolide (BR) plays a key role in the regulation of abiotic stress responses in plants. However, the effects of brassinolide on alleviating Zn phytotoxicity in watermelon (*Citrullus lanatus* L.) seedlings are not clear. The purpose of this study was to study the effect of 24-epibrassinolide (EBR, one of the bioactive BRs) on Zn tolerance of watermelon seedlings and its potential resistance mechanism. Exposure to excessive Zn significantly inhibited shoot and root fresh weight of watermelon, but this could be significantly alleviated by the optimum 0.05 μM EBR. Exogenous spraying EBR increased the pigments and alleviated oxidative damage caused by Zn through reducing Zn accumulation and the levels of reactive oxygen species (ROS) and malonaldehyde (MDA) and increasing the activities of antioxidant enzymes and contents of ascorbic acid (AsA) and glutathione (GSH). Importantly, the relative mRNA levels of antioxidant genesincluding *Cu/Zn-superoxidedismutase* (*Cu-Zn SOD*), *catalase* (*CAT*), *ascorbic acid peroxidase* (*APX*), and *glutathione reductase* (*GR*) were significantly induced after EBR treatment. In addition, EBR pre-treatment induced lignin accumulation under Zn stress, and the activity of phenylalanine ammonia-lyase (PAL) and 4-coumaric ligase (4CL), two key enzymes regulating lignin synthesis, also tended to be consistent. Collectively, the present research proves the beneficial effects of EBR in response to Zn stress through enhancing antioxidant defense and lignin accumulation and provides a new insight into the mechanism of BR-enhancing heavy metal tolerance.

## Introduction

Zinc (Zn) is an essential trace element for crop growth. Zn has no redox activity, but it plays a structural and/or catalytic role in many processes (Broadley et al., 2007; Sagardoy et al., 2009). Recently, due to the accelerated urbanization, continuous use of Zn fertilizer, mining and smelting activities, and industrial wastewater, the toxicity of Zn in plants has become a concern, which is reflected in the increase of Zn content in surface soil (Ramakrishna & Rao, 2015; Kaur & Garg, 2021). The increased concentration of Zn is toxic to plants (Broadley et al., 2007; Sagardoy et al., 2009; Ren et al., 2023). Potentially harmful Zn levels in soil may lead to various changes of plants, such as inhibition of growth, decrease of photosynthesis and respiration rates, imbalance of mineral nutrient, and producing reactive oxygen species (ROS) (Kaur & Garg, 2021; Wei et al., 2022). Excessive production of ROS can cause oxidative damage to nucleic acid, protein, cell membrane, *etc*., and change the antioxidant protection system, eventually leading to cell death (Michael & Krishnaswamy, 2011; Kaur & Garg, 2021; Zhang & Liao, 2021; Jabeen et al., 2022).

The application of plant growth regulator is considered to be an important way to assuage the injurious effects of heavy metal (Li et al., 2022). Brassinolide is the first active brassinosteroids isolated. It is internationally recognized as the most active and broad-spectrum plant growth hormone known as the sixth plant hormone (Haubrick & Assmann, 2006). BRs can stimulate the internal potential of plants, promote crop growth, improve the antioxidant performance under drought, low/high temperature, heavy metals, and other adverse environments, reduce membrane lipid peroxidation, and promote photosynthesis and dry matter accumulation, thus enhancing plant stress tolerance (Basit et al., 2021). Epibrassinolide (EBR) is a synthetic brassinolide analogue, which has been widely used in production. For example, exogenous EBR improves cold induced oxidative stress in grape seedlings (Chen et al., 2019). Under low temperature stress, EBR can reduce oxidative stress, increase osmoregulation substances, and improve the photosynthesis of winter radish rape (Li et al., 2021). EBR promotes the activation of physiological compensation mechanism under drought stress and water replenishment improving soybean yield (Silva et al., 2022). EBR also plays a positive role in *Colletotrichum fructicola* resistance by inducing lignin synthesis (Zhang et al., 2022). BRs can be used as multi-dimensional regulators for plants to respond to various environmental stresses, which has been reviewed by Basit et al. (2021).

In addition, more and more studies show that EBR can significantly enhance plant tolerance to heavy metals such as cadmium (Hasan et al., 2008; Niu et al., 2023), copper (Zhou et al., 2018), manganese (Surgun-Acar & Zemheri-Navruz, 2022), nickel (Maia et al., 2022), and Zn (Ramakrishna & Rao, 2015; Wu et al., 2016; Ren et al., 2023). For example, exogenous EBR spraying increases the tolerance of eggplant seedlings to Zn stress through the enhancement of antioxidant enzyme activity, osmotic substance accumulation, and hormone metabolism balance (He et al., 2016). Recently, EBR application to *M. sativa* could reduce the accumulation of Zn, increase antioxidant defense response, and actively regulate the detoxification of heavy metal (Ren et al., 2023). Although the signal mechanism of BRs regulating plant growth has been established, the mechanism of BRs affecting tolerance to environmental stress is still unclear. No one has reported the relationship between BRs induced heavy metal resistance of plants and lignin synthesis.

In China, vegetable production has been faced with the risk of heavy metal pollution, including Zn (He et al., 2016). Watermelon (*Citrullus lanatus*) is one of the most important horticultural crops. However, so far, the possibility of using EBR to improve the Zn tolerance of watermelon has not been studied, let alone the regulatory mechanism. Therefore, the purpose of this study was to evaluate whether exogenously application of EBR could increase Zn tolerance in watermelon, and if so, what is the possible physiological mechanism of function mediated by EBR? We first screened the optimal spray concentration of EBR for Zn tolerance, then studied its influences of EBR on photosynthetic pigment contents, lipid peroxidation, total antioxidant level, activities of antioxidant enzymes, the relative expression levels of antioxidant related genes, Zn accumulation, and lignin synthesis in watermelon seedlings exposed to Zn stress.

## Materials and Methods

### Plant materials and growth conditions

Watermelon (*C. lanatus* L.) variety 8,424 was from Anhui Fengsheng Agricultural Technology Co., Ltd. Watermelon seeds were sterilized, washed, placed on filter article, and incubated at 25 °C. After seed germination, the seedlings were sown in pots (diameter of 8 cm), which was filled with sterilized sand and vermiculite (3:1). Growth conditions: temperature 25 ± 2 °C, relative humidity 70 ± 5%, photoperiod 12/12 h, photosynthetic photon flux density 500 μmol m^−2^ s. After 10 days, seedlings were irrigated with 1/2 Hoagland’s nutrient.

### Stress treatments

The Zn (in the form of ZnSO_4_·7H_2_O) concentration was screened based on the preliminary experiment, using 0, 2.5, 5.0, or 10.0 mM of Zn and 5 mM Zn was selected (Fig. S1). The EBR concentrations were screened using 0.025, 0.05, 0.10, 0.20, or 0.50 μM EBR and 0.05 μM EBR were selected based on the growth index. EBR was dissolved in ethanol to obtain the stock solution, and then dilute it in pure water to obtain the working concentration of EBR.

At the 4-leaf stage, healthy seedlings with similar growth were randomly divided into two groups: control and Zn treatment group. For EBR concentration screening, the following experimental design was carried out: plants were subjected to Zn and EBR-free nutrient solution (Control), or subjected to 5.0 mM Zn with different concentration of EBR (0, 0.025, 0.05, 0.10, 0.20, or 0.50 μM EBR). Seedlings were sprayed with EBR 1 day in advance and watered with the nutrient containing Zn every 3 days. After 10 days of Zn/EBR application, all seedlings were harvested and the fresh weight was recorded.

For physiological mechanism research, the following experimental design was carried out: (1) plants were subjected to Zn and EBR-free nutrient solution (Control); (2) plants were subjected to Zn alone (Zn); (3) plants were subjected to 0.05 μM EBR alone (Zn); (4) plants were subjected to both Zn and EBR (Zn+EBR). After 10 days of Zn/EBR treatment, the third leaves were frozen in liquid N_2_ and kept at −80 °C until analysis.

### Photosynthetic pigments

Photosynthetic pigments (chlorophyll a, chlorophyll b) were determined using 0.1 g of fresh leaf extract. The leaf extract was obtained by holding the leaves in 10 ml of 80% frozen acetone for 24 h. The extract was then at 4 °C for 10,000 g centrifuge. The following formula was used to record the absorbance:

Chlorophyll a = 12.7 OD663– 2.69 OD645

Chlorophyll b = 22.9 OD645– 4.68 OD663

### Hydrogen peroxide and lipid peroxidation

As described by Ding, Zhang & Qin (2015), 3, 3′-diaminobenzidine (DAB) staining was used to detect the generation of hydrogen peroxide (H_2_O_2_). DAB staining solution (0.1 mg/ml DAB, 1% isopropanol and 0.1% Triton X-100) was used to immerse the fresh leaves in the dark at 28 °C for 12 h. In order to remove the chlorophyll after DAB staining, the leaves were cultured in 70% ethanol solution, then photographed. H_2_O_2_ and MDA contents were measured according to the previous method of Wu, Xia & Zou (2006). Briefly, the extraction mixture was prepared by homogenizing 0.1 g of fresh plant leaves in 3 ml of 5% (w/v) trichloro acetic acid (TCA) and centrifuged at 12,000×g for 15 min. To measure H_2_O_2_, 0.2 ml of supernatant and 0.9 ml of reaction mixture (2.5 mM potassium phosphate buffer (pH 7.0) and 500 mM potassium iodide) were mixed, and 390 nm was used to record the absorbance. For MDA, 1.0 ml of supernatant was mixed with 1.0 ml of the reaction mixture, incubated in boiling water for 30 min, and centrifuged at 10,000×g for 10 min. The MDA content was measured at 600 nm and 532 nm using a spectrophotometer.

### Enzyme activities

To measure the activities of antioxidant enzymes, fresh leaves (0.5 g) were quickly homogenized in liquid N_2_ and suspended in 5 ml 50 mM PBS (pH 7.5) containing 1 mM EDTA, 2% PVP. After centrifugation at 15,000×g and 4 °C for 30 min, the supernatant fraction was used in the following enzyme activity assays according to the method of Ding et al. (2018) and Maia et al. (2022). Superoxide dismutase (SOD, E.C. 1.15.1.1) activity was measured through its ability to inhibit the photochemical reduction of nitroblue tetrazolium (NBT). One unit of SOD activity is the amount of enzyme required to cause 50% inhibition of the reduction rate of NBT as monitored at 560 nm. Peroxidase (POD; E.C. 1.11.1.7) activity was determined by monitoring the absorbance change caused by the oxidation of guaiacol at 470 nm using H_2_O_2_. Enzyme activity was quantified by the amount of tetraguaiacol formed using its molar extinction coefficient (ε = 26.6 mM^−1^ cm^−1^). Catalase (CAT; E.C. 1.11.1.6.) activity was determined by measuring the consumption of H_2_O_2_ at 240 nm. Enzyme activity was quantified by the amount of H_2_O_2_ consumed using its molar extinction coefficient (ε = 45.2 mM^−1^ cm^−1^). The activity of ascorbic acid peroxidase (APX; E.C.1.11.1.11) was recorded as the decrease of absorbance at 290 nm of ascorbate and enzyme activity was quantified using the molar extinction coefficient for 2.8 mM^−1^ cm^−1^. Glutathione reductase (GR; E.C.1.6.4.2) activity was determined according to the rate of glutathione-dependent oxidation of NADPH at 340 nm (ε = 6.22 mM^−1^ cm^−1^).

Phenylalanine ammonia-lyase (PAL) and 4-coumaric ligase (4CL) are two key lignin biosynthetic enzymes (Shao et al., 2022). PAL and 4CL activities were measured by ELISA kit (JiangLai, Shanghai, China) following the protocol. PAL catalyzes the decomposition of L-phenylalanine to trans-cinnamic acid and ammonia. Trans-cinnamic acid has a maximum absorption value at 290 nm. The PAL activity was calculated by measuring the absorbance rate. 4CL catalyzes 4-coumaric acid and CoA to produce 4-coumaric acid CoA. The formation rate of 4-coumaric acid CoA at 333 nm can reflect the activity of 4CL.

### Antioxidant content

According to the method of Qiu et al. (2014), 0.5 g of fresh leaves were ground in 1 ml of precooled 0.5 M sodium phosphate buffer solution (PH 7.8) and centrifuged, then 10% trichloroacetic acid (TCA) of equal volume was added for AsA determination, or 10% sulfosalicylic acid was added for GSH determination. ASA was determined by 512 nm spectrophotometry with supernatant after adding ascorbic acid oxidase. AsA content was calculated based on a standard curve of AsA. For the determination of glutathione (GSH), the amount of GSH is calculated by the change of absorbance at 412 nm after adding 5-50-dithiobis (2-nitrobenzoic acid) (DTNB). The reduced GSH contents were calculated based on a standard curve of GSH.

### Gene expression analysis

Total RNA from watermelon leaves was isolated and the SuperscriptIII first strand synthesis system (Invitrogen, Shanghai, China) was used for cDNA synthesis following the manufacturer’s protocol. Use SYBR Premium Ex Taq (Takara, Dalian, China) to conduct qRT-PCR in qRT-PCR system. The primers of *Cu–Zn SOD, CAT, APX*, and *GR* were designed according to the previous studies (Mo, Yang & Liu, 2016; Ye et al., 2019) based on the Watermelon Genome Database (http://www.icugi.org), shown in Table S1.

### Quantification of Zn and lignin content

The dried material were weighed, ground to a powder, and digested with a 1:3 mixture of HCl:HNO3. The digests were then dissolved in ultrapure water. Then, the digested sample were analyzed using a flame-atomic absorption spectrometer (AAS; PerkinElmer, Waltham, MA, USA) and the contents were expressed as mg g^−1^ dry weight. The lignin content of leaves was determined by ultraviolet spectrophotometry according to the method of Xu et al. (2022) using a lignin content determination kit (COMINBIO, Suzhou, China). Dry the sample at 80 °C to constant weight, grind, sift, weigh about 5 mg into 1.5 ml EP tube, and then follow the steps in the instruction. Acetyl lignin is produced after the phenol hydroxyl in lignin is acetylated. The product has a characteristic absorption peak at 280 nm. The lignin content was measured on the basis of the changing absorbance.

### Statistical analysis

Three biological replicates were set for each experimental treatment. The data is the average ± SD (Standard Deviation) of the replicates displayed by the vertical error bar. One-way analysis of variance (ANOVA) and the Least Significance Difference (LSD) test (significance level is 0.05, *P* value ≤0.05) were used to analyze the difference between the experimental groups (Jabeen et al., 2022). SPSS version 20.0 (IBM, Armonk, NY, USA) was used for statistical analysis.

## Results

### Effects of EBR on the watermelon growth under Zn stress

The dose-dependent responses of watermelon to EBR under 5 mM Zn stress at six levels (0, 0.025, 0.05, 0.10, 0.20, or 0.50 μM) were first evaluated with respect to shoot and root fresh weight. When compared to the control group, Zn treatment significantly decreased shoot and root fresh weight by 21.8% and 44.8%, respectively (Table 1). Under Zn stress, pre-treatment with 0.025 and 0.05 μM EBR significantly improved shoot fresh weight, while only 0.05 μM EBR obviously improved root fresh weight. Compared with the Zn treatment, 0.05 μM EBR (Zn+EBR2 treatment) increased shoot and root fresh weight by 14.4% and 30.8%, respectively. Therefore, 0.05 μM EBR was selected as a beneficial dose for the following physiological mechanism analysis.

**Table 1:** Effect of different concentration of EBR on watermelon growth under Zn stress.

Treatment	Control	Zn	Zn+EBR1	Zn+EBR2	Zn+EBR3	Zn+EBR4	Zn+EBR5
Shoot fresh weight (g)	5.52 ± 0.52a	4.32 ± 0.29c	4.96 ± 0.20b	4.94 ± 0.15b	4.25 ± 0.20c	4.11 ± 0.30cd	3.72 ± 0.19d
Root fresh weight (g)	1.76 ± 0.13a	0.97 ± 0.05c	1.08 ± 0.11c	1.27 ± 0.03b	1.01 ± 0.06c	0.84 ± 0.04d	0.71 ± 0.02e

**Note:**

The data is the average ± SD of the three replicates displayed by the vertical error bar. Different letters in each line indicate that there is a significant difference between them (*P* ≤ 0.05). Plants were subjected to Zn and EBR-free nutrient solution (Control), or subjected to 5.0 mM Zn with different concentration of EBR (0, 0.025, 0.05, 0.10, 0.20, or 0.50 μM EBR).

### Effects of EBR on the chlorophyll content under Zn stress

**Figure 1:**
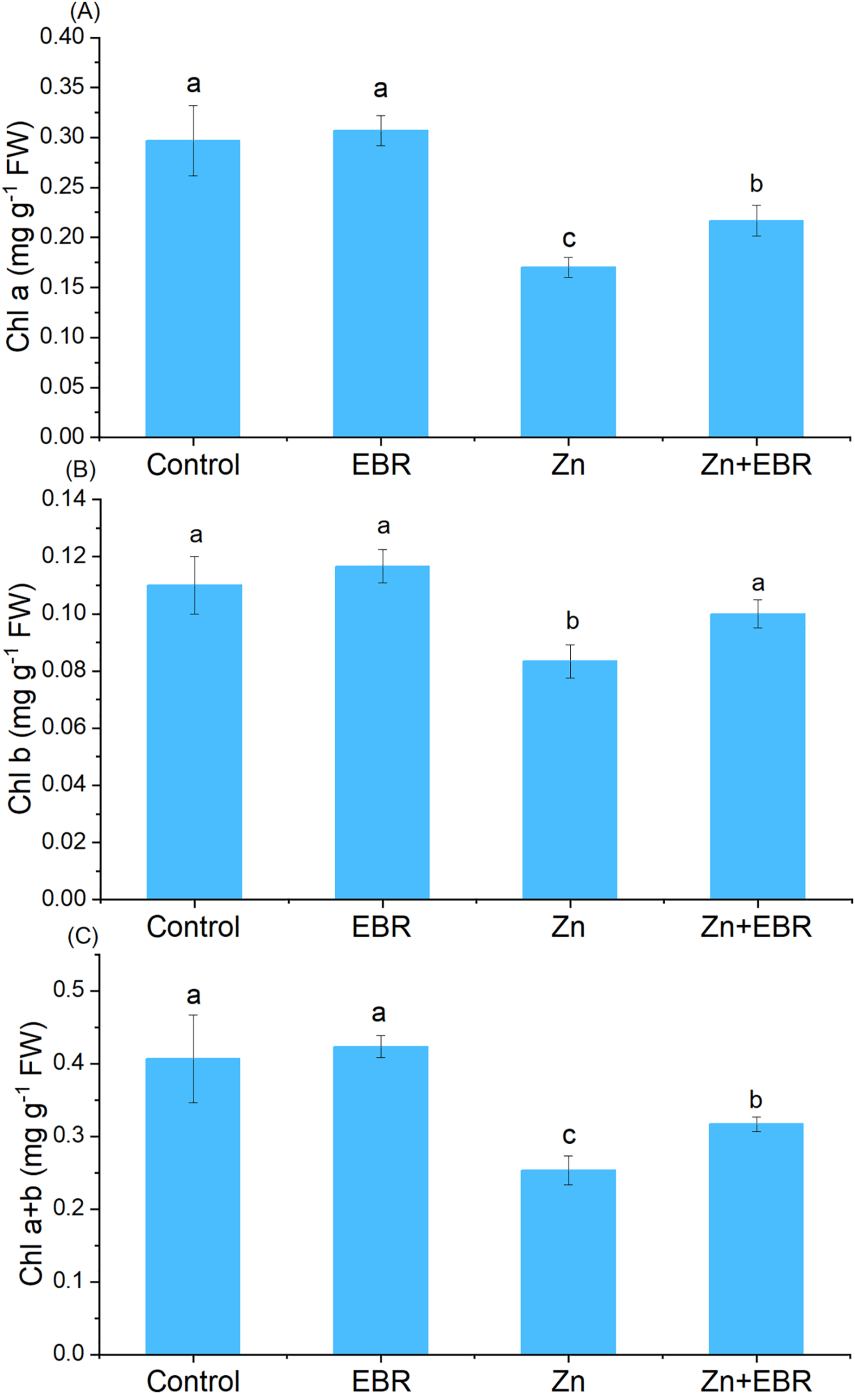
Fffects of EBR on the levels of chlorophyll a (A), chlorophyll b (B), and chlorophyll a+b (C) in watermelon seedling leaves under Zn stress. The data is the average ± SD of the three replicates displayed by the vertical error bar. According to the LSD test, different letters indicate that there are significant differences between them (*P* ≤ 0.05). (1) Plants were subjected to Zn- and EBR-free nutrient solution (Control); (2) plants were subjected to 0.05 μM EBR alone (Zn); (3) plants were subjected to Zn alone (Zn); (4) plants were subjected to both Zn and EBR (Zn+EBR). The notes in the following Figures are the same as those in this description.

Compared to the control group, Zn treatment significantly reduced chlorophyll content (Fig. 1). Nonetheless, EBR treatment led to the reversal of this effect, and the levels of chlorophyll a, chlorophyll b and chlorophyll a+b increased (by 27.5%, 20.0%, and 25.0% over Zn treatment). Under Zn-free condition, exogenous EBR slightly improved chlorophyll level, but the difference was not significant.

### Effects of EBR on the contents H_2_O_2_ and MDA under Zn stress

**Figure 2:**
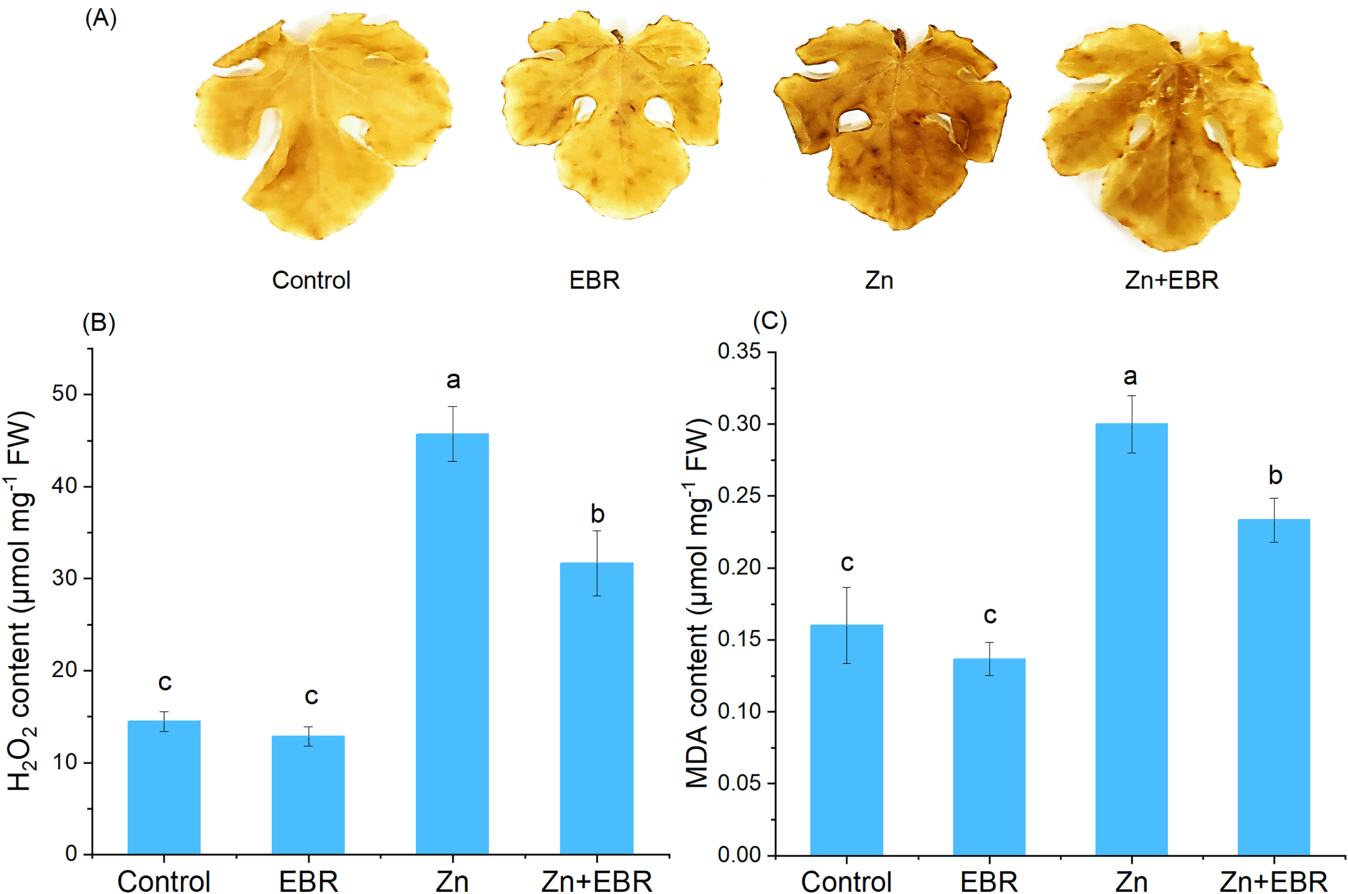
Effects of EBR on H_2_O_2_ production (A, B) and MDA level (C) in the leaves of watermelon seedlings under Zn stress.

Zn stress caused the increase of H_2_O_2_ content in leaves, confirmed by histochemical analysis and H_2_O_2_ quantification (Figs. 2A and 2B). As expected, compared with the control plants, the leaves treated with Zn showed more obvious spots, but EBR pre-treatment significantly reduced the dyeing intensity (Fig. 2A). Quantitative analysis of H_2_O_2_ showed the same trend (Fig. 2B), indicating that EBR spraying pre-treatment reduces ROS accumulation induced by Zn stress. To further investigate the mitigation of EBR on Zn-induced oxidative stress in watermelon, we measured MDA content. MDA content in leaves was 87.5% higher than that in control after Zn stress (Fig. 2C). EBR-pretreated plants showed significantly lower MDA content (by 22.2% over Zn treatment).

### Effects of EBR on the antioxidant enzymatic activities under Zn stress

**Figure 3:**
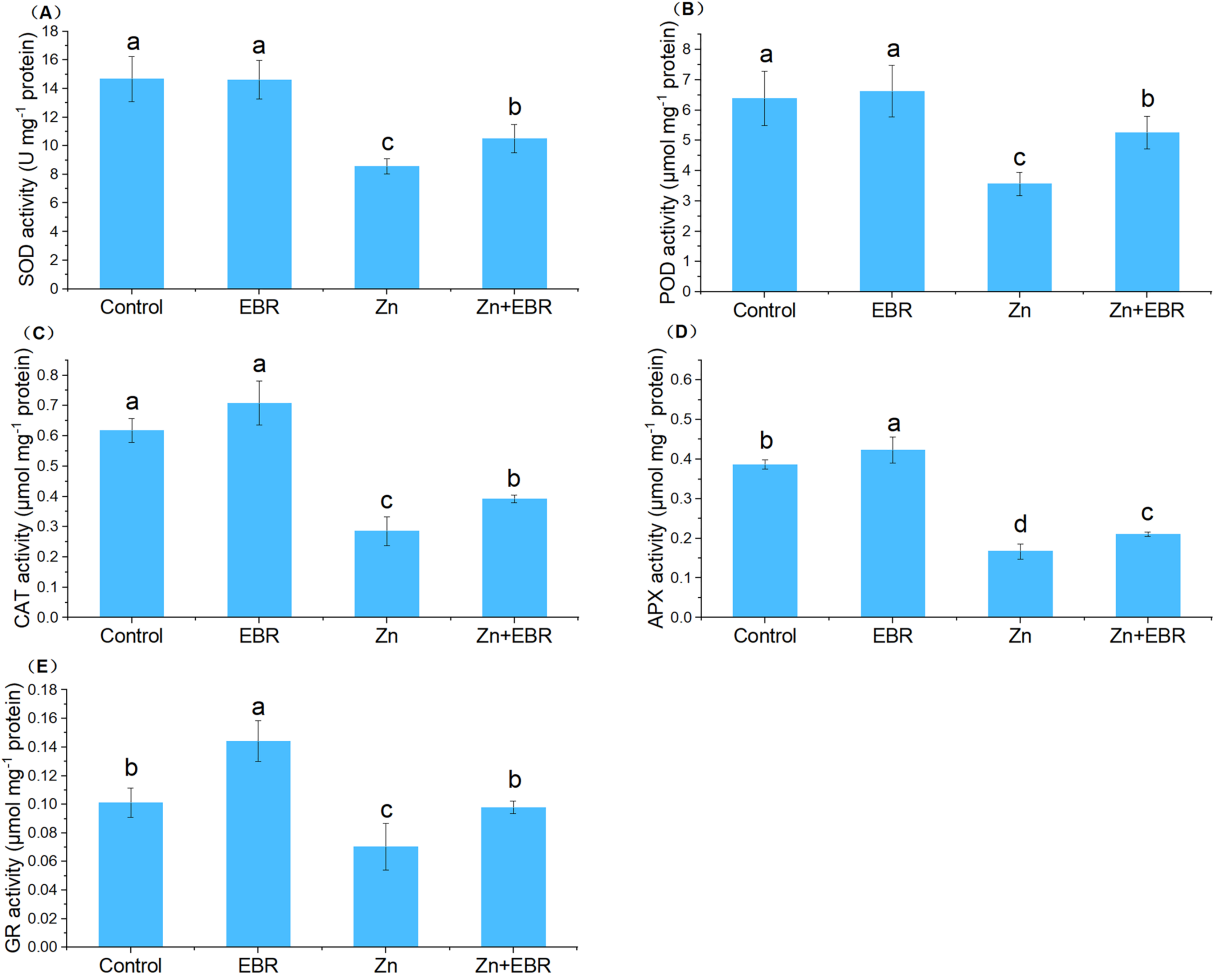
Effects of EBR on the activities of superoxide dismutase (SOD) (A), guaiacol peroxidase (POD) (B), catalase (CAT) (C), ascorbate peroxidase (APX) (D), and glutathione reductase (GR) (E) in the leaves of watermelon seedlings under Zn stress.

In order to adapt to oxidative damage caused by ROS, plants have evolved an antioxidant protection system, which is composed of enzymes that scavenge ROS like SOD, POD, CAT, APX, and GR, to maintain the homeostasis of cell redox within a specific threshold (Zhang & Liao, 2021). The activities of SOD, POD, CAT, and APX were significantly decreased under Zn treatment (Fig. 3). Exogenous application of EBR to the stressed plants restrained this downward trend in these enzymatic activities. Compared to Zn stress alone, EBR enhanced these enzymatic activities by 22.6%, 47.3%, 37.0%, and 26.2%, respectively under Zn stress. Without Zn stress, EBR treatment had no significant effects on these enzyme activities. As far as GR enzyme activity is concerned, it presents a different response mode from other enzymes. Under normal conditions, a high level of GR activity was observed in EBR-pretreated plants. The GR activity of watermelon plants poisoned by Zn decreased significantly. The application of EBR to plants in response to Zn stress caused an increase in GR activity. The activity of GR increased by 39.0% in EBR-pretreated plants compared with those under Zn stress (Fig. 3), which was fully restored to the control level. These results suggested that EBR pre-treatment of watermelon seedlings improved watermelon tolerance to Zn stress through the regulation of antioxidant enzymatic activities.

### Effects of EBR on the contents of AsA and GSH under Zn stress

**Figure 4:**
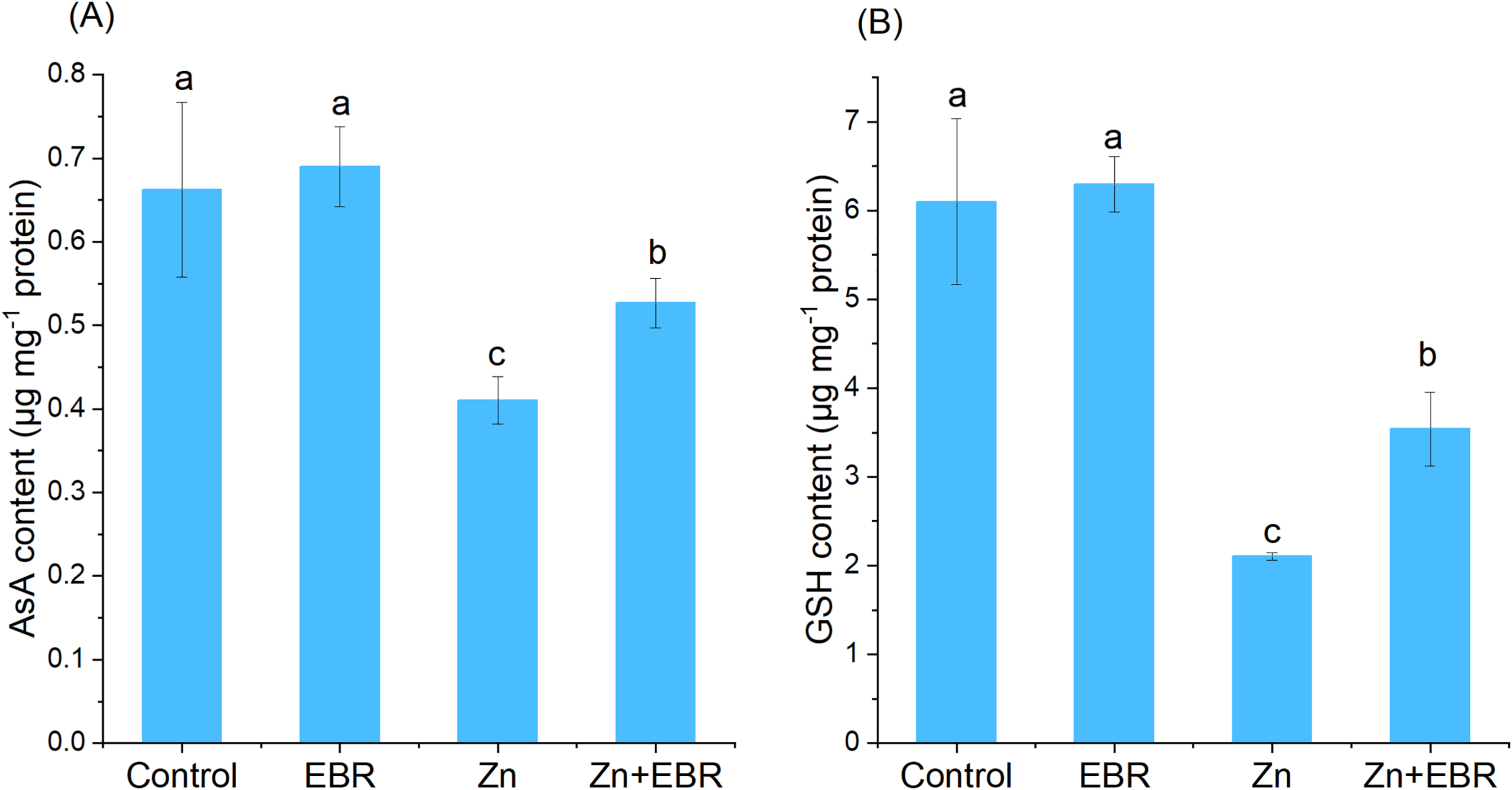
Effects of EBR on the levels of ascorbic acid (AsA) (A) and reduced glutathione (GSH) (B) in the leaves of watermelon seedlings under Zn stress.

AsA and GSH are non-enzymatic antioxidants, maintaining the balance between the generation and elimination of ROS. When compared to the control, the contents of AsA and GSH in watermelon leaves significantly decreased. Under Zn stress (Fig. 4). This trend was reversed in Zn-stressed plants pretreated with EBR. Compared with Zn-exposed plants without EBR, EBR increased the contents of AsA and GSH (28.4% and 68.1%, respectively).

### Effects of EBR on the expression profiles of antioxidant enzymatic genes under Zn stress

**Figure 5:**
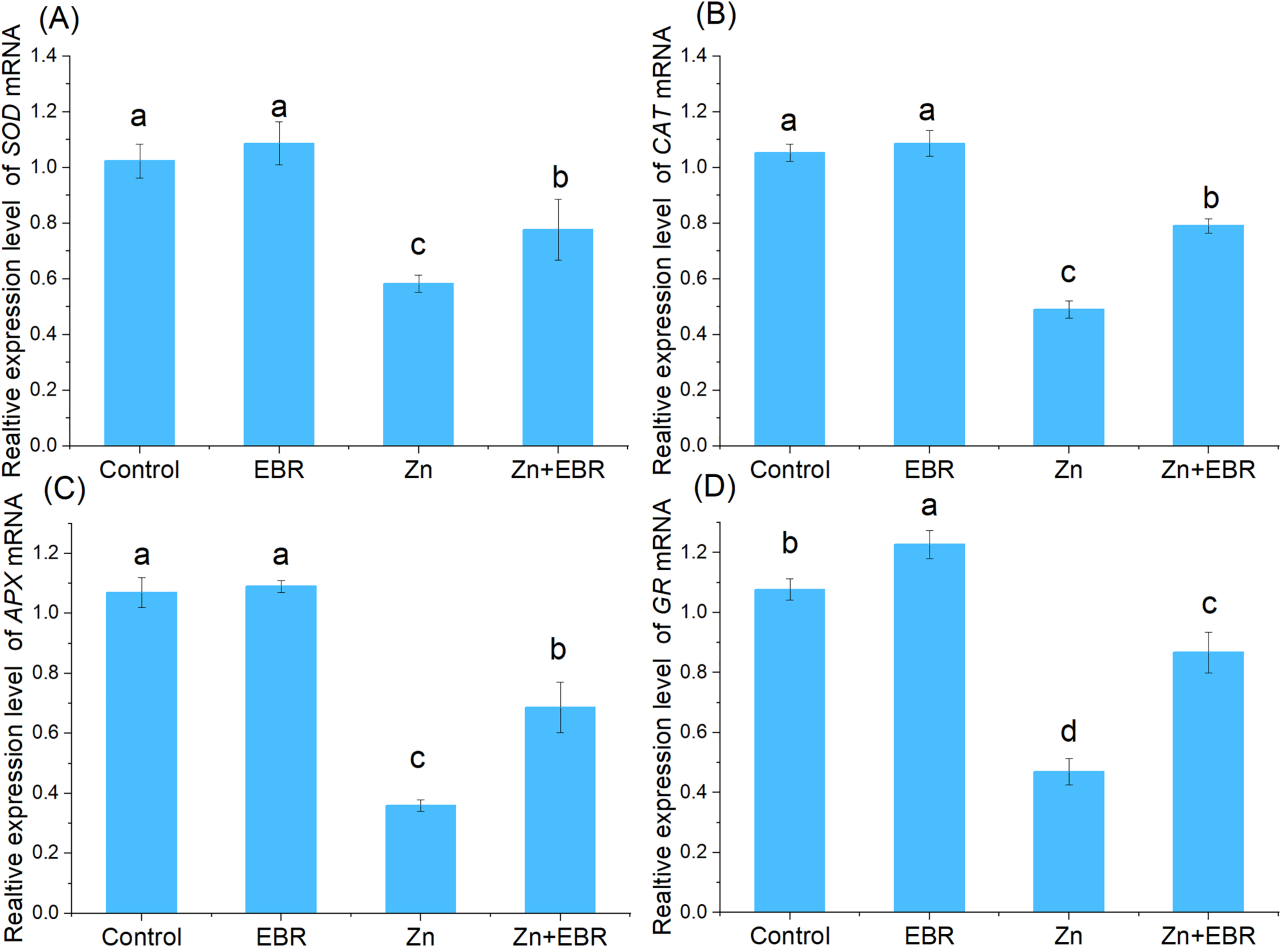
Relative expression levels of *SOD* (A), *CAT* (B), *APX* (C), and *GR* (D) genes in the leaves of watermelon seedlings under Zn stress.

In order to understand the molecular regulation of EBR-treated watermelon seedlings under Zn stress, the relative expression of mRNA of antioxidant genes (*Cu-Zn SOD, CAT, APX, GR*) was analyzed. As shown in Fig. 5, compared with the control condition, the relative expression of antioxidant enzyme gene was down—regulated after Zn treatment. This inhibition was significantly reduced after pre-treatment with EBR. There was no significant difference in gene expression between normal and EBR alone treatment (Fig. 5). The result suggested that EBR could significantly increase the expression of antioxidant genes under Zn stress, then regulate the antioxidant responses.

### Effects of EBR on the contents of lignin and Zn under Zn stress

**Figure 6:**
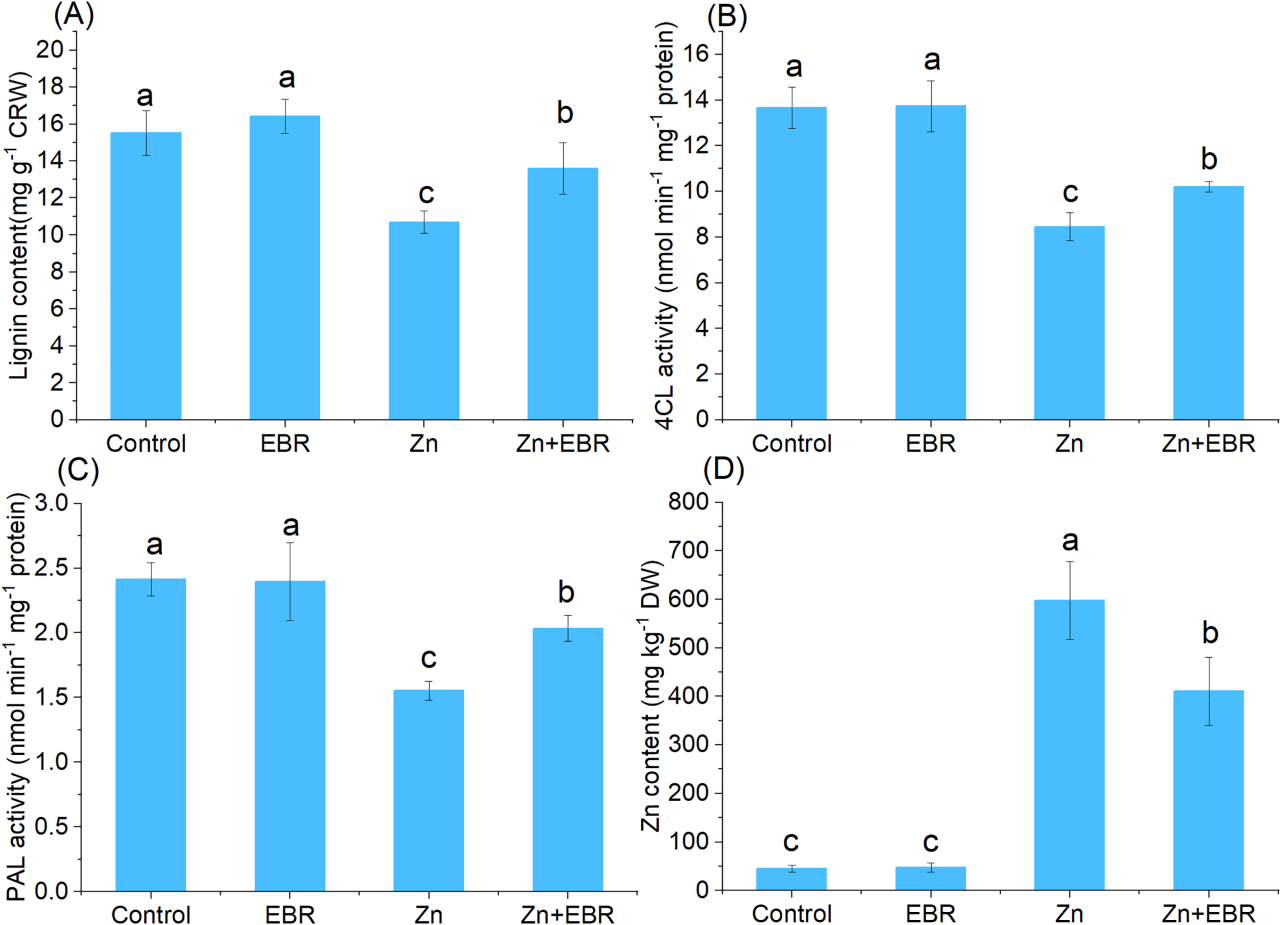
Effects of EBR on the content of Zn and lignin in the leaves of watermelon seedlings under Zn stress. (A) Lignin content; (B) 4CL activity; (C) PAL activity; (D) Zn content.

To determine whether EBR could tolerate Zn stress by regulating lignin accumulation, the lignin content of leaves was also determined. As shown in Fig. 6A, increasing Zn level in growth media significantly decreased the lignin content, but EBR pre-treatment significantly increased lignin content. 4CL and PAL are two key lignin biosynthetic enzymes (Shao et al., 2022), which were determined. Zn remarkably decreased 4CL and PAL activities compared to control, but EBR pre-treatment restrained this trend (Figs. 6B and 6C), which was consistent with the change trend of the index of POD activity studied above (Fig. 3B). These results indicated that EBR increased lignin content by promoting the activities of key enzymes in charge of lignin synthesis.

EBR induced watermelon tolerance to Zn stress accompanied by a decrease in Zn accumulation. As expected, compared with the control plant, the leaves treated with Zn showed a large amount of Zn accumulation, but EBR pre-treatment significantly reduced Zn accumulation (Fig. 6D).

## Discussion

EBR is a BR molecule with the strongest biological activity, which plays an essential role in the process of plant development and the regulation of stress responses in plants (Tanveer et al., 2018; Basit et al., 2021). This effect is dose dependent, and the appropriate concentration of EBR varies with plant species, experimental conditions, and application methods. For example, the most effective response was at 0.01 μM EBR for wheat growth under salt stress (Dong et al., 2017) and *Brassica juncea* under high temperature (Sirhindi et al., 2009); 0.2 μM EBR for *Hordeum vulgare* under salt and potassium deficiency (Liaqat et al., 2020); 0.75 μM EBR for *S. lineare* under Zn stress (Zhang & Liao, 2021). So far, the role of BRs in reducing zinc toxicity of watermelon has not been studied. In this study, EBR could significantly reduce the inhibition of watermelon growth caused by Zn in a dose-dependent manner, and 0.05 μM of EBR would be the optimum concentration for increasing Zn tolerance in watermelon seedlings (Table 1), showing that EBR could protect watermelon plants from Zn toxicity and participate in the regulation of the response of watermelon to Zn stress. The results are similar to those of earlier studies, *i.e.*, pre-spraying 0.1 μM EBR can significantly reduce the Zn induced toxicity of eggplant seedlings under Zn stress (He et al., 2016; Wu et al., 2016). BRs can respond to stress stimulation and growth and development in many ways, such as regulating antioxidant system, protecting photosynthesis, and interacting with hormones (Hu, Wei & Liao, 2021).

One of the most obvious harmful symptoms of toxic heavy metals is the reduction of photosynthetic pigments (Wei et al., 2022). Excess Zn can interfere with iron and magnesium homeostasis, chlorophyll biosynthesis and protein composition of photosynthetic membrane, and eventually lead to chlorophyll degradation (Ramakrishna & Rao, 2015). Here, Zn stress significantly reduced the chlorophyll levels, while EBR pre-treatment increased these levels (Fig. 1). The results were consistent with the results of He et al. (2016), who found that EBR increased the chlorophyll content of eggplant seedlings in response to Zn stress. Ramakrishna & Rao (2012) reported that the foliar application of EBR alleviated Zn toxicity on radish, mainly protected the chloroplast membrane, and increased chlorophyll a, chlorophyll b and Car. The increase of chlorophyll content induced by EBR is because that BRs may directly or indirectly stimulate chlorophyll biosynthesis or inhibit chlorophyllase activity (Hayat et al., 2011). Besides, EBR-induced increase in the levels of chlorophyll under stress was related to the decrease of ROS accumulation, thus reducing oxidative damage to thylakoid membrane structure and function (Ramakrishna & Rao, 2012; Maia et al., 2022). The possible antioxidant mechanism for the beneficial effects of EBR application is discussed in the following sections.

Zn does not directly participate in the production of ROS, but its toxicity can indirectly participate in the production of high levels of ROS (Lin & Aarts, 2012). Overproduction of ROS can lead to macromolecular oxidative damage, irreparable metabolic dysfunction and cell death (Kaur & Garg, 2021; Zhang & Liao, 2021; Jabeen et al., 2022). Therefore, we predicted that the protective effect of EBR might be associated with the balance of ROS metabolism. The current results showed that EBR could reduce Zn-induced oxidative damage, which was evidenced by the reduction of H_2_O_2_ and MDA levels. The present results agree with the previous studies, that are, BRs treatment can reduce the lipid peroxidation caused by heavy metals, such as cadmium (Hasan et al., 2008), copper (Zhou et al., 2018), and nickel (Maia et al., 2022). Scavenging ROS is essential to alleviate oxidative stress under environmental stress and maintain normal plant metabolism. Plants have developed enzymatic and non-enzymatic defense systems to combat ROS-induced oxidative damage (Farooq et al., 2019). SOD is a major superoxide radical scavenger. Its activity leads to the formation of H_2_O_2_ and water, becoming the first line of defense. Subsequently, H_2_O_2_ is removed by CAT and several peroxidases (Laxa et al., 2019). After the treatment with Zn, the activities of SOD, CAT, APX, GR, and POD were increased in watermelon plants pretreated with EBR (Fig. 3). These results were consistent with Ramakrishna & Rao (2015), who reported that EBR increased the activities of SOD, POD, CAT, APX and GR of *R. sativus* under Zn stress. In the same mode, EBR increased the levels of antioxidant system of grape cuttings (Zhou et al., 2018) under copper stress and *Arabidopsis thaliana* under manganese stress (Surgun-Acar & Zemheri-Navruz, 2022). Non-enzyme antioxidants AsA and GSH maintain the redox state of cells by acting as substrates in the AsA-GSH cycle, thus providing cell protection (Sharma & Dubey, 2005). In this cycle, APX uses AsA to detoxify H_2_O_2_. GSSG is converted back to GSH by NADPH dependency GR. In this study, EBR application to Zn-stressed seedlings increased APX and GR activities (Fig. 3) and AsA and GSH levels (Fig. 4), indicating that BRs can balance ROS level by regulating AsA and GSH cycles of watermelon plants. The result was in agreement with a previous report, where foliar spraying of BRs greatly enhanced ASA and GSH contents in *Raphanus sativus* under Zn stress (Ramakrishna & Rao, 2015). Wu et al. (2016) pointed out that EBR up-regulated the activities of GR, GS, DHAR and MDHAR to promote the regeneration of GSH and AsA in eggplant seedlings under Zn stress. Therefore, it is indicated that exogenous EBR sprayed could detoxify ROS by making AsA-GSH cycle run at a higher rate to reduce lipid peroxidation and maintain the internal stability of cell redox, and thus improve the tolerance of watermelon seedlings to Zn stress.

The enhanced antioxidant defense system seems to be the result of the activation or increase of related gene transcription, which increases the resistance of plants to oxidative stress under zinc stress (He et al., 2016). It has been proved that EBR increases the expression of *CAT* and *cAPX* in cucumber (Xia et al., 2009). The co-exposure of EBR and Mn resulted in further increase of SOD and CAT enzyme activities and gene expression of *copper/zinc superoxide dismutase 3* (*CSD3*), *iron superoxide dismutase 2* (*FSD2*), *iron superoxide dismutase 3* (*FSD3*), and *catalase 2* (*CAT2*) in *Arabidopsis thaliana* (Surgun-Acar & Zemheri-Navruz, 2022). In this study, to better understand the increase of antioxidant enzyme activity induced by EBR, the effects of Zn and EBR on the relative expression of key antioxidant enzyme genes was studied. After EBR treatment, the relative expression levels of antioxidant related genes *Cu-Zn SOD, CAT, APX*, and *GR* in Zn-treated seedlings significantly increased (Fig. 5). Sharma, Kumar & Bhardwaj (2016) showed similar results; that is, under the influence of EBR, the antioxidant enzyme activity and the expression level of these genes (*M. SOD, Cu/Zn SOD, Cat A, Cat B, APX*, and *GR*) in rice seedlings under Cr ions significantly increased (Sharma, Kumar & Bhardwaj, 2016). Here, the activities of antioxidant enzymes and the gene expression of antioxidant genes were found to be increased in plants inoculated with EBR under Zn stress, indicating that EBR induces an effective ROS scavenging mechanism to protect watermelon plants from oxidative damage caused by Zn.

Lignin is a natural physical barrier against heavy metal (Berni et al., 2019). It has been reported that EBR mediates the synthesis of lignin and then promotes the ability of plants to resist biological stress, but there are few reports on heavy metal stress. For example, EBR plays a positive role in *Colletotrichum fructicola* resistance through the induction of lignin synthesis (Zhang et al., 2022). It was speculated that EBR might help to reduce the Zn toxicity in watermelon through this pathway. In this study, EBR pre-treatment increased lignin content, which was consistent with the activities of PAL and 4CL, playing a key role in lignin biosynthesis (Shao et al., 2022). PAL is the first-step and rate-limiting enzyme, which catalyzes the conversion of phenylalanine to trans-cinnamic acid. Then, C4H and 4CL catalyze the conversion of cinnamic acid to p-coumarinoyl-CoA, which is the precursor of phenols and lignin. Phenols have antioxidant characteristics participating in ROS removal and form metal complexes to protect plants from abiotic stress (Sharma et al., 2019). The increased amount of lignin in the secondary cell wall, which is closely related to Zn absorption and transport, thus making EBR-treated plants more tolerant. The deposition of “stress lignin” is indeed a protective mechanism against pathogen entry and abiotic stress (Berni et al., 2019). All these results suggested that lignin synthesis was an important module involved in EBR-induced Zn tolerance.

## Conclusion

In summary, the results have clearly demonstrated that Zn stress causes severe oxidative stress and significantly decreases photosynthetic pigments leading to plant growth inhibition. Exogenous pre-spraying EBR regulates watermelon Zn tolerance in a dose-dependent manner, and 0.05 μM was the optimum concentration. The application of EBR can enhance the tolerance of watermelon seedlings to Zn stress by regulating antioxidants and improving enzyme activity and gene expression mode of antioxidant system, reducing H_2_O_2_ content and lipid peroxidation to protect against oxidative stress, thus improving watermelon tolerance to Zn stress to some extent. In addition, EBR enhances the biosynthesis of cell wall *e.g.*, lignin content, improve the efficiency of cell wall Zn fixation, and thus reduce the toxicity of Zn. These results provide important clues for understanding the defense mechanism of EBR in response to Zn stress and provide a new insight into the mechanism of BR-enhancing heavy metal tolerance.

## Supplemental Information

10.7717/peerj.15330/supp-1 Supplemental Information 1 Sequences of primers for qRT-PCR.

10.7717/peerj.15330/supp-2 Supplemental Information 2 Effects of different Zn concentrations on the growth of watermelon seedlings.

10.7717/peerj.15330/supp-3 Supplemental Information 3 Raw data.

## Additional Information and Declarations

### Data Availability

The following information was supplied regarding data availability: The raw data is available in the Supplemental Files.
