# Supplementary material for: 24-Epibrassinolide confers zinc stress tolerance in watermelon seedlings through modulating antioxidative capacities and lignin accumulation
Source: PeerJ. 2023 May 9;11:e15330. doi: 10.7717/peerj.15330 (PMC10178286; doi:10.7717/peerj.15330)
Supplement: Supplemental Information 1 [file peerj-11-15330-s001.docx]

**Table S1.** Sequences of primers for qRT-PCR.

| Gene | Primer sequence (5’-3’) | Gene description |
| --- | --- | --- |
| Cu-Zn SOD | F: AGCCATTGTAGATACCCAGATTC | Cu-Zn subunit-superoxide dismutase |
|  | R: CTGAGTTCGTGACCTCCTTT |  |
| CAT | F: ACTTGTGCCGATTTCCTTCG | Catalase |
|  | R: ATTGCCCTCCCTGGTGTAA |  |
| APX | F: GGGAAGTTGAACGGATTAGAG | Cytoplasmic ascorbate peroxidase |
|  | R: CAGCATCGTGAAATACCAGAC |  |
| GR | F: GGAGTCGCTGTCGCTCTTAA | Cytoplasmic glutathione reductase |
|  | R: CTACCTGCTTCAATTCGCCT |  |
|  |  |  |
