# Supplementary figures and images for: 24-Epibrassinolide confers zinc stress tolerance in watermelon seedlings through modulating antioxidative capacities and lignin accumulation
Source: PeerJ. 2023 May 9;11:e15330. doi: 10.7717/peerj.15330 (PMC10178286; doi:10.7717/peerj.15330)

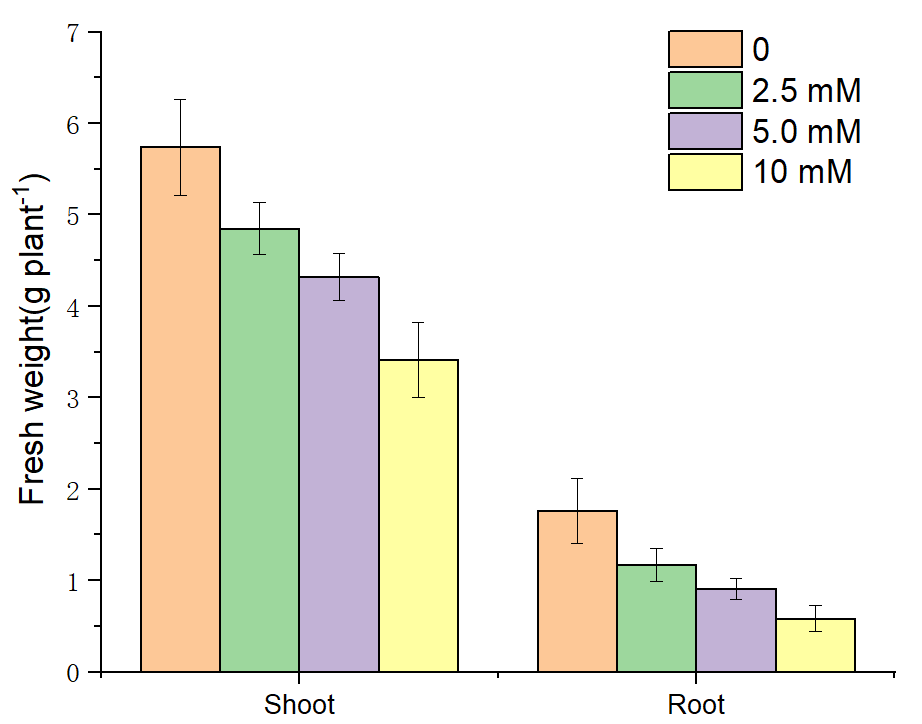


Figure S1 Effects of different Zn concentrations on the growth of watermelon seedlings

Supplement: Supplemental Information 2 [file peerj-11-15330-s002.docx]
